# Gap analysis between trainees' subjective competencies and the competencies expected by instructors in urology: A need assessment survey in Japan

**DOI:** 10.1111/iju.15430

**Published:** 2024-02-17

**Authors:** Kanta Hori, Takashige Abe, Noriyuki Abe, Junya Abe, Kazufumi Okada, Keita Takahashi, Shigeru Harada, Jun Furumido, Sachiyo Murai, Masafumi Kon, Kohei Hashimoto, Naoya Masumori, Hidehiro Kakizaki, Nobuo Shinohara

**Affiliations:** ^1^ Department of Urology Hokkaido University Graduate School of Medicine Sapporo Japan; ^2^ Department of Urology Asahikawa Medical University Asahikawa Japan; ^3^ Department of Urology Sapporo Medical University Sapporo Japan; ^4^ Promotion Unit, Data Science Center, Institute of Health Science Innovation for Medical Care Hokkaido University Hospital Sapporo Japan

**Keywords:** gap analysis, needs assessment survey, surgical education, surgical training, urologic surgical procedures

## Abstract

**Objective:**

According to the rapid progress in surgical techniques, a growing number of procedures should be learned during postgraduate training periods. This study aimed to clarify the current situation regarding urological surgical training and identify the perception gap between trainees' competency and the competency expected by instructors in Japan.

**Methods:**

Regarding the 40 urological surgical procedures selected via the Delphi method, we collected data on previous caseloads, current subjective autonomy, and confidence for future skill acquisition from trainees (<15 post‐graduate years [PGY]), and the competencies when trainees became attending doctors expected by instructors (>15 PGY), according to a 5‐point Likert scale. In total, 174 urologists in Hokkaido Prefecture, Japan were enrolled in this study.

**Results:**

The response rate was 96% (165/174). In a large proportion of the procedures, caseloads grew with accumulation of years of clinical practice. However, trainees had limited caseloads of robotic and reconstructive surgeries even after 15 PGY. Trainees showed low subjective competencies at present and low confidence for future skill acquisition in several procedures, such as open cystectomy, ureteroureterostomy, and ureterocystostomy, while instructors expected trainees to be able to perform these procedures independently when they became attending doctors.

**Conclusion:**

Trainees showed low subjective competencies and low confidence for future skill acquisition in several open and reconstructive procedures, while instructors considered that these procedures should be independently performable by attending doctors. We believe that knowledge of these perception gaps is helpful to develop a practical training program.

**Abbreviations**
JUA: Japanese Urological Association
NB: neobladder reconstruction
PCNL: percutaneous nephrolithotripsy
PGY: post‐graduate years
RAPN: robot‐assisted partial nephrectomy
RARC: robot‐assisted radical cystectomy
RARP: robot‐assisted radical prostatectomy
RPLND: retroperitoneal lymph node dissection

## INTRODUCTION

According to the accelerating progress in new surgical techniques and novel surgical devices, such as laparoscopic and robot‐assisted surgery, a growing number of procedures should be learned during post‐graduate training periods, including urological resident training. However, due to working‐hour restrictions and medical safety, young trainees now face difficulty in accumulating sufficient surgical caseloads during training periods. Several previous studies involving general surgery revealed that trainees did not achieve sufficient surgical competencies after their residency periods.^1^, ^2^, ^3^ In urology, similar trends have also been reported, for example, in the United States, a survey of US urology residents/recent graduates and program directors revealed a lack of confidence in performing unsupervised advanced laparoscopic and robotic surgeries in most residents.^4^ A national survey in Italy also observed that more than 70% of residents had never performed a robotic procedure during training.^5^

To date, no study has determined the current status of urological surgical training in Japan. Hokkaido is the second largest island in Japan, and more than five million people live there. There are three medical universities, and over 200 urologists conduct daily clinical practice. In the present study, in order to clarify the current situation regarding urological training in Japan, we performed a needs assessment survey involving both urology trainees and instructors and aimed to identify perception gaps between trainees' subjective competencies and competencies expected by instructors.

## METHODS

This research was approved by the Institutional Review Board (No. 020‐035). As mentioned above, in Hokkaido, there are three medical schools (Hokkaido University, Sapporo Medical University, and Asahikawa Medical University), and their affiliated hospitals. Excluding those who were not regularly involved in post‐graduate surgical training, all urologists practicing at these hospitals were invited to participate in the present study by e‐mail. After obtaining written informed consent regarding the use of their data for research, we started data collection with either web‐based (SurveyMonkey®) or paper‐based questionnaires.

### Development of core surgical procedure list

Before data collection, based on the 3‐round Delphi method, we selected “40 core urological surgical procedures” out of the 121 procedures listed in a case‐log book for a board‐certified urologist by the Japanese Urological Association (JUA). Briefly, we invited 15 attending doctors. Eleven of the doctors were working at Hokkaido University Hospital, and their subspecialities were: uro‐oncology (*n* = 5), kidney transplantation (*n* = 2), pediatric urology (*n* = 2), and voiding dysfunction/urinary reconstruction (*n* = 2). The remaining 4 doctors were working at our teaching hospitals (Obihiro Kosei Hospital: *n* = 1, Kushiro City General Hospital: *n* = 1, and Otaru General Hospital: *n* = 2). Their clinical experiences ranged from 13 to 41 years. We then asked them about their expected competencies based on a 5‐point Likert scale when trainees became attending urologists regarding the 121 procedures [Round 1: a response rate of 100% (15/15)]. Of the 67 procedures achieving above a mean of 3 points in Round 1, 26 procedures were eliminated at the time of the web meeting [Round 2: an attendance rate of 100% (15/15)] mainly due to similarities among the procedures and rare indications in real‐world clinical practice, and 3 procedures achieving below a mean of 3 points were added again based on the attending doctors' opinions. After the final anonymous vote asking them if each procedure should be included in the list of core surgical procedures, out of the 44 candidates, the 40 procedures which more than 80% of the respondents agreed to include were selected [Round 3: a response rate of 87% (13/15)]. Table 1 shows a list of 40 core procedures selected. We divided them into 8 categories for subsequent analysis (transurethral, stone, pediatric, laparoscopic, open, reconstructive, robotic surgery, and others).

**TABLE 1:** List of the 40 core procedures selected after the Delphi method.

No.	Procedures	Abbreviation	Category
1	Transurethral resection of bladder tumor	TURBT	Transurethral
2	Transurethral electrocoagulation	TUC	Transurethral
3	Transurethral resection of prostate	TURP	Transurethral
4	Double‐J catheterization	Double‐J	Stone
5	Transurethral cystolithotripsy	TUCL	Stone
6	Percutaneous nephrostomy	Nephrostomy	Stone
7	Transurethral ureterolithotripsy	TUL	Stone
8	Percutaneous nephrolithotripsy	PNL	Stone
9	Circumcision	Circumcision	Pediatric
10	Orchidopexy (testicular torsion)	Orchidopexy (torsion)	Pediatric
11	Orchidopexy	Orchidopexy	Pediatric
12	Laparoscopic radical nephrectomy	L‐Nephrectomy	Laparoscopic
13	Laparoscopic adrenalectomy	L‐adrenalectomy	Laparoscopic
14	Laparoscopic nephroureterectomy	L‐nephroureterectomy	Laparoscopic
15	Open radical nephrectomy	O‐Nephrectomy	Open
16	Open cystostomy	O‐cystostomy	Open
17	Open partial nephrectomy	O‐partial nephrectomy	Open
18	Open nephroureterectomy	O‐nephroureterectomy	Open
19	Open total cystectomy	O‐Cystectomy	Open
20	Open prostatectomy	O‐Prostatectomy	Open
21	Open retroperitoneal lymph node dissection	O‐RPLND	Open
22	Ileal (colon) conduit (with total cystectomy)	Ileal conduit	Reconstructive
23	Cutaneous ureterostomy (with total cystectomy)	Cutaneous ureterostomy	Reconstructive
24	Ureterocystostomy	Ureterocystostomy	Reconstructive
25	Open ureteroureterostomy	Ureteroureterostomy	Reconstructive
26	Open neobladder	Neobladder	Reconstructive
27	Open pyeloplasty	PYELOPLASTY	Reconstructive
28	Open Boari flap	Boari flap	Reconstructive
29	Robotic‐assisted laparoscopic radical prostatectomy	RARP	Robot
30	Robotic‐assisted laparoscopic partial nephrectomy	RAPN	Robot
31	Robot‐assisted radical cystectomy	RARC	Robot
32	Orchidectomy	Orchidectomy	Others
33	Percutaneous cystostomy	Cystostomy	Others
34	High orchidectomy	High orchidectomy	Others
35	Testicular hydrocele repair	Hydrocele	Others
36	Total urethrectomy	Total urethrectomy	Others
37	Vascular Access Construction	Vascular access	Others
38	Partial penectomy	Partial penectomy	Others
39	Transvaginal tape/transobturator tape	TVT/TOT	Others
40	Varicocele repair	Varicocele	Others

### Survey

Our survey comprised two parts. In the first part, we asked participants about their backgrounds (age, sex, years after graduation from medical university, credentials, specialty, previous caseloads of open, laparoscopic, robotic, and transurethral surgery, etc.), hospital information (number of beds, number of urologists, number of robots, annual case volume under general anesthesia, availability of training simulators, etc.), and other current situations regarding surgical education. In the second part, regarding the 40 core surgeries abovementioned, we collected data on previous caseloads, current subjective autonomy, and confidence for future skill acquisition from participants with less than 15 years of clinical experience (referred to as “trainees”). In terms of subjective autonomy, the 5‐point Dreyfus scale was utilized. The Dreyfus scale is a simple scale which evaluates competency to perform a procedure according to five levels.^6^

⮚ Level 1. Show and tell: The instructor performs the critical step while explaining it to the learner.
⮚ Level 2. Active help: The instructor actively guides the learner through the critical step of the procedure.
⮚ Level 3. Passive help: The learner performs the critical step independently while the instructor passively provides skilled assistance and intervenes only when necessary for an important teaching point or to optimize patient safety.
⮚ Level 4. Supervision only: Instructor presence is necessary only to guarantee patient safety. At this level, the learner has enough proficiency to perform the step independently with a less‐skilled assistant, while the instructor does not need to be directly involved in the procedure other than to provide close supervision.
⮚ Level 5. Expert: Learner performs procedures and manages complications independently.

From participants with more than 15 years of clinical experience (referred to as “instructors”), we collected expectations of competencies for each procedure when trainees become attending urological doctors, according to a Likert scale (from very low to very high expectation on a 5‐point Likert scale). From both groups, we collected data on assessment of potential risk if the procedure is performed by a noncompetent surgeon (from very low to very high on a 5‐point Likert scale). Tables [Link], [Link] show the details of the questionnaire (original Japanese and English‐translated versions).

The survey was delivered in February 2021 (Hokkaido University and Asahikawa Medical University Groups), and in August 2021 (Sapporo Medical University Group) via e‐mail. Each URL link (for trainees or instructors) to SurveyMonkey® was separately sent to each group. Data collection was open for 60 days, with one e‐mail reminder sent to all candidates, with an additional mail/e‐mail reminder sent to non‐responders. In this paper, we present the participants' backgrounds and results of the second part.

### Analysis

For each procedure, means ± standard deviations were calculated according to each question. In terms of previous caseloads of “trainees,” the proportion giving each answer was evaluated. Furthermore, in the present study, we focused on the following analyses:

✓ Analysis 1: Trainees' caseloads of each procedure divided by post‐graduate years (PGY).
✓ Analysis 2: Trainees' self‐assessment of autonomy for each procedure at present.
✓ Analysis 3: Instructors' expected competency versus trainees' confidence for future skill acquisition when the trainee becomes an attending urologist.
✓ Analysis 4: Instructors' versus trainees' assessment of potential risk if the procedure is performed by an inexperienced surgeon.

In terms of Analysis 3, we performed hierarchical cluster analysis to examine the recognition gap between two groups. Hierarchical analysis with the ward method was conducted to create a dendrogram and scatter plot to visually determine the reasonable number of clusters. We also performed sub‐analyses after dividing “trainees” into a junior group (3–5 PGY), intermediate (6–10 PGY), and senior (11–15 PGY) when necessary. All statistical analyses were completed using JMP Pro version 16.0.0.

## RESULTS

A total of 174 urologists were invited to participate in this study, and the response rate was 96% (167/174). Table 2 summarizes the backgrounds of participants. Table S5 summarizes the results for each question for the 40 core procedures.

**TABLE 2:** Summary of participants' backgrounds.

Variables	Trainees, *n* = 85	Instructors, *n* = 82
Age years, median (range)	32 (27–43)	50 (40–65)
Sex		
Male	72 (84.7)	80 (97.6)
Female	13 (15.3)	2 (2.4)
Post‐graduate years, median (range)	6 (3–15)	25 (16–41)
–5 years	35 (41.2)	
6–10 years	34 (40.0)	
11–15 years	16 (18.8)	
16– years	0	82 (100)
Certification/credentials		
Board‐certified urologists	33 (38.8)	82 (100)
Attending doctor authorized by Japanese Urological Association	15 (17.6)	82 (100)
Endoscopic surgical skill qualification	9 (10.6)	53 (64.6)
Robot surgery certification	39 (45.9)	46 (56.0)
Number of surgeries attended as primary operator		
Open surgeries		
0 cases	13 (15.3)	0 (0)
1–10 cases	21 (24.7)	0 (0)
11–50 cases	33 (38.8)	3 (3.7)
51–100 cases	10 (11.8)	8 (9.8)
101–500 cases	8 (9.4)	42 (51.2)
501 cases	0 (0)	29 (35.4)
Laparoscopic surgeries		
0 cases	17 (20)	6 (7.3)
1–10 cases	18 (21.2)	4 (4.9)
11–50 cases	38 (44.7)	12 (14.6)
51–100 cases	9 (10.6)	10 (12.2)
101–500 cases	3 (3.5)	42 (51.2)
501 cases	0 (0)	8 (9.8)
Robotic surgeries		
0 cases	50 (58.8)	33 (40.2)
1–10 cases	12 (14.1)	7 (8.5)
11–50 cases	18 (21.2)	6 (7.3)
51–100 cases	4 (4.7)	8 (9.8)
101–500 cases	1 (1.2)	27 (32.9)
501 cases	0 (0)	1 (1.2)
Transurethral surgeries		
0 cases	0 (0)	0 (0)
1–10 cases	1 (1.2)	0 (0)
11–50 cases	7 (8.2)	0 (0)
51–100 cases	13 (15.3)	1 (1.2)
101–500 cases	46 (54.1)	22 (26.8)
501 cases	18 (21.2)	59 (72)

### Analysis 1: Trainees' caseloads of each procedure divided by PGY

**FIGURE 1:**
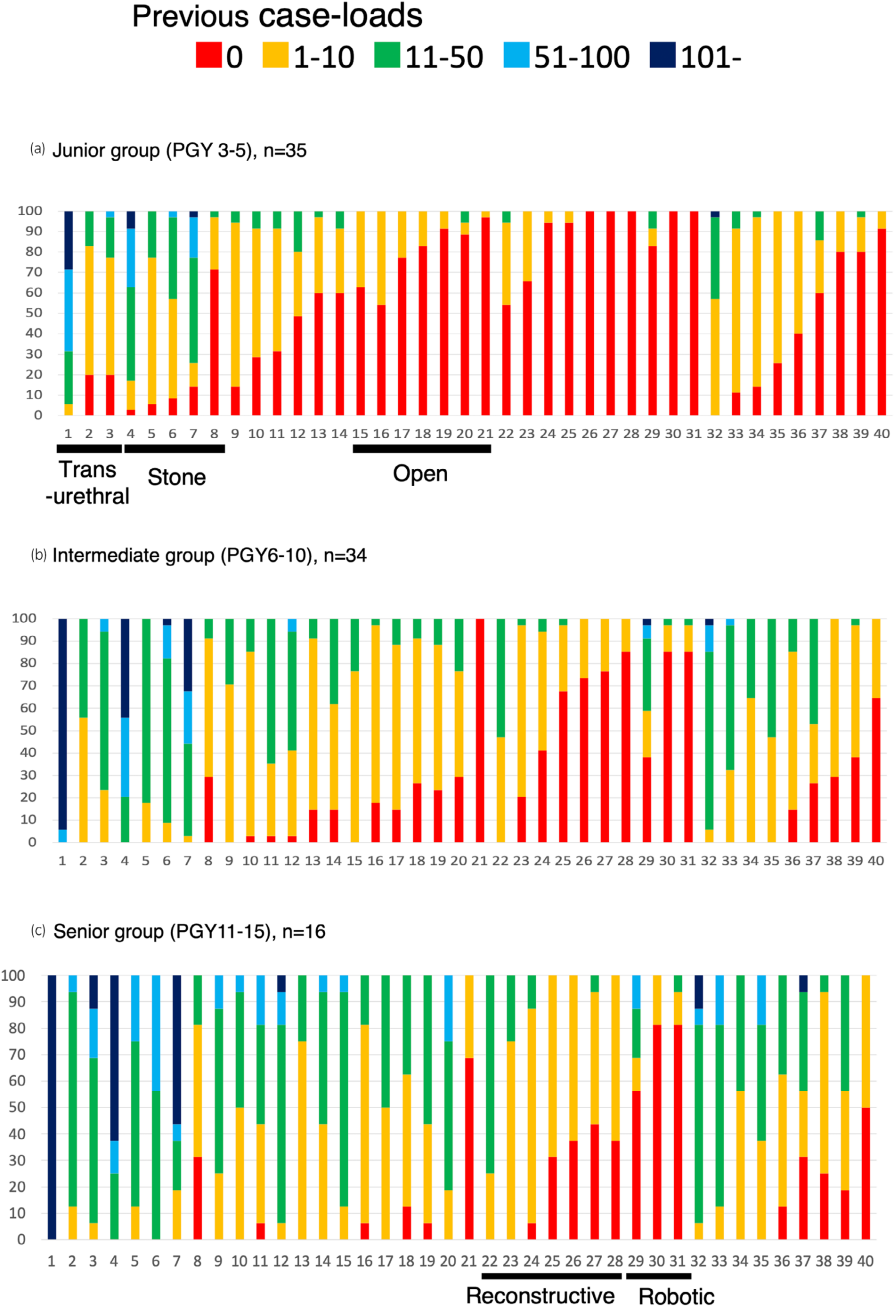
Summary of trainees' previous caseloads for 40 core procedures divided by post‐graduate years (PGY). (a) Junior group (PGY 3‐5), (b) Intermediate group (PGY 6‐10), and (c) Senior group (PGY 11‐15). For a large proportion of the procedures, caseloads grew with accumulation of years of clinical practice. However, regarding reconstructive (No. 22–28) and robotic (No. 29–31) surgeries, the senior participants still had limited caseloads, other than ileal conduit (No. 22).

Figure 1 summarizes caseloads for each procedure, divided by PGY (junior: *n* = 35, intermediate: *n* = 34, and senior group: *n* = 16). For a large proportion of procedures, caseloads grew with accumulation of years of clinical practice. The junior participants had some experience in transurethral (No. 1–3) and stone (No. 4–7) surgeries, other than percutaneous nephrolithotripsy (PCNL, No. 8). Regarding reconstructive (No. 22–28) and robotic (No. 29–31) surgeries, the senior participants still had limited caseloads, other than ileal conduit (No. 22). In terms of open surgeries (No. 15–21), the junior group had limited surgical experience, while the intermediate and senior groups had growing surgical experience (No. 15–20), other than open retroperitoneal lymph node dissection (RPLND, No. 21).

### Analysis 2: Trainees' self‐assessment of autonomy for each procedure at present

**FIGURE 2:**
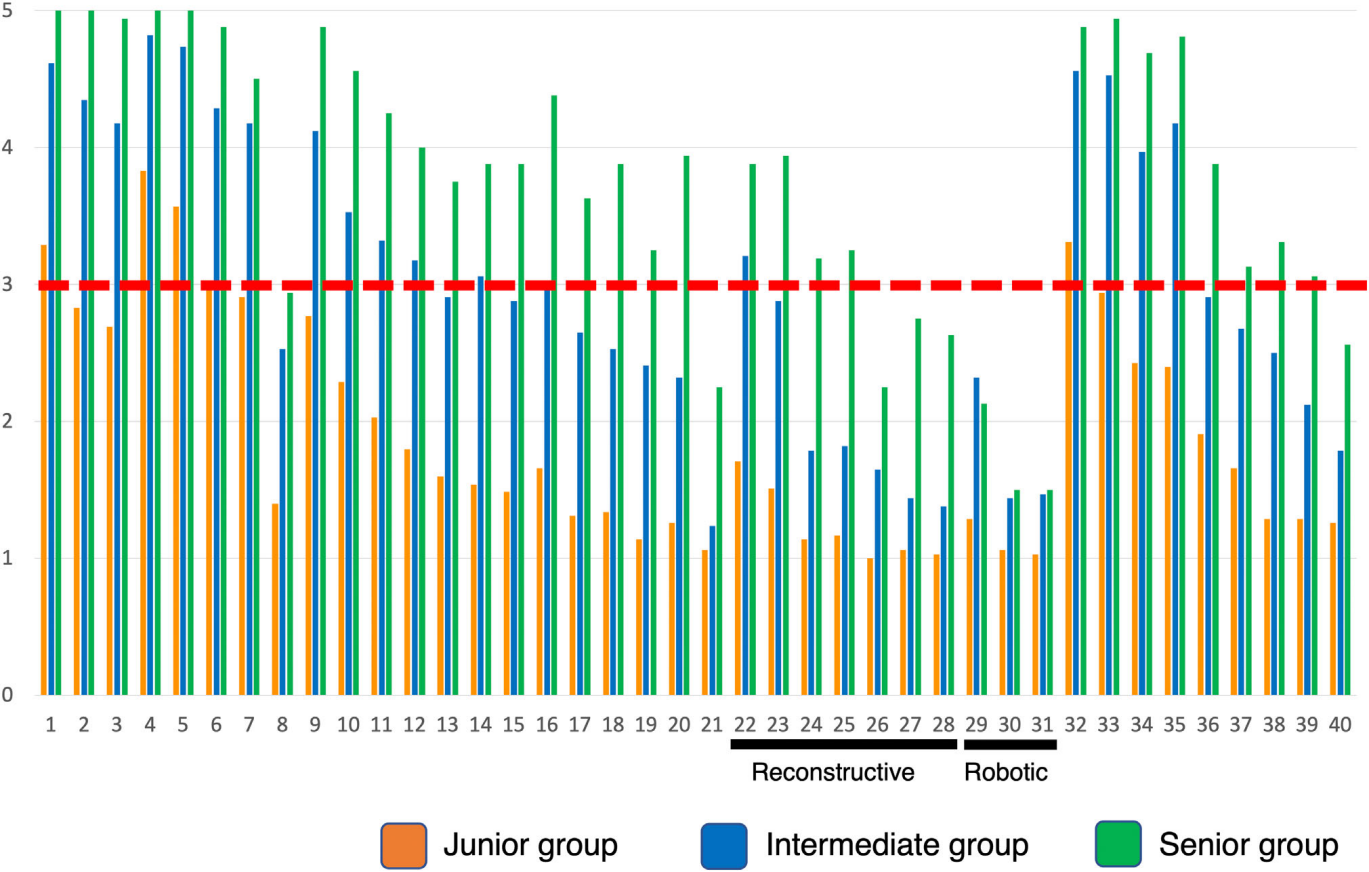
Summary of mean score of trainees' self‐assessment of surgical autonomy divided by post‐graduate years. Overall, for a large proportion of the procedures, self‐assessed surgical autonomy grew with accumulation of years of clinical practice, other than percutaneous nephrolithotripsy (No. 8), retroperitoneal lymph node dissection (No. 21), open neobladder construction (No. 26), open pyeloplasty (No. 27), open Boari flap (No. 28), robot‐assisted radical prostatectomy (No. 29), robot‐assisted partial nephrectomy (No. 30), robot‐assisted radical cystectomy (No. 31), and varicocele repair (No. 40).

Figure 2 shows a summary of trainees' self‐assessment of surgical autonomy divided by PGY. Overall, for a large proportion of the procedures, self‐assessed surgical autonomy grew with accumulation of years of clinical practice. However, in terms of PCNL (No. 8), open RPLND (No. 21), open neobladder reconstruction (NB, No. 26), open pyeloplasty (No. 27), open Boari‐flap (No. 28), robot‐assisted radical prostatectomy (RARP, No. 29), robot‐assisted partial nephrectomy (RAPN, No. 30), robot‐assisted radical cystectomy (RARC, No. 31), and varicocele repair (No. 40), self‐assessed surgical autonomy remained low‐level. These procedures did not reach a mean score of 4 in Q4 (feasibility of training in a corresponding procedure) for instructors (Table S5).

### Analysis 3: Instructors' expected competency versus trainees' assessment of confidence for future skill acquisition when the trainee becomes an attending urologist

**FIGURE 3:**
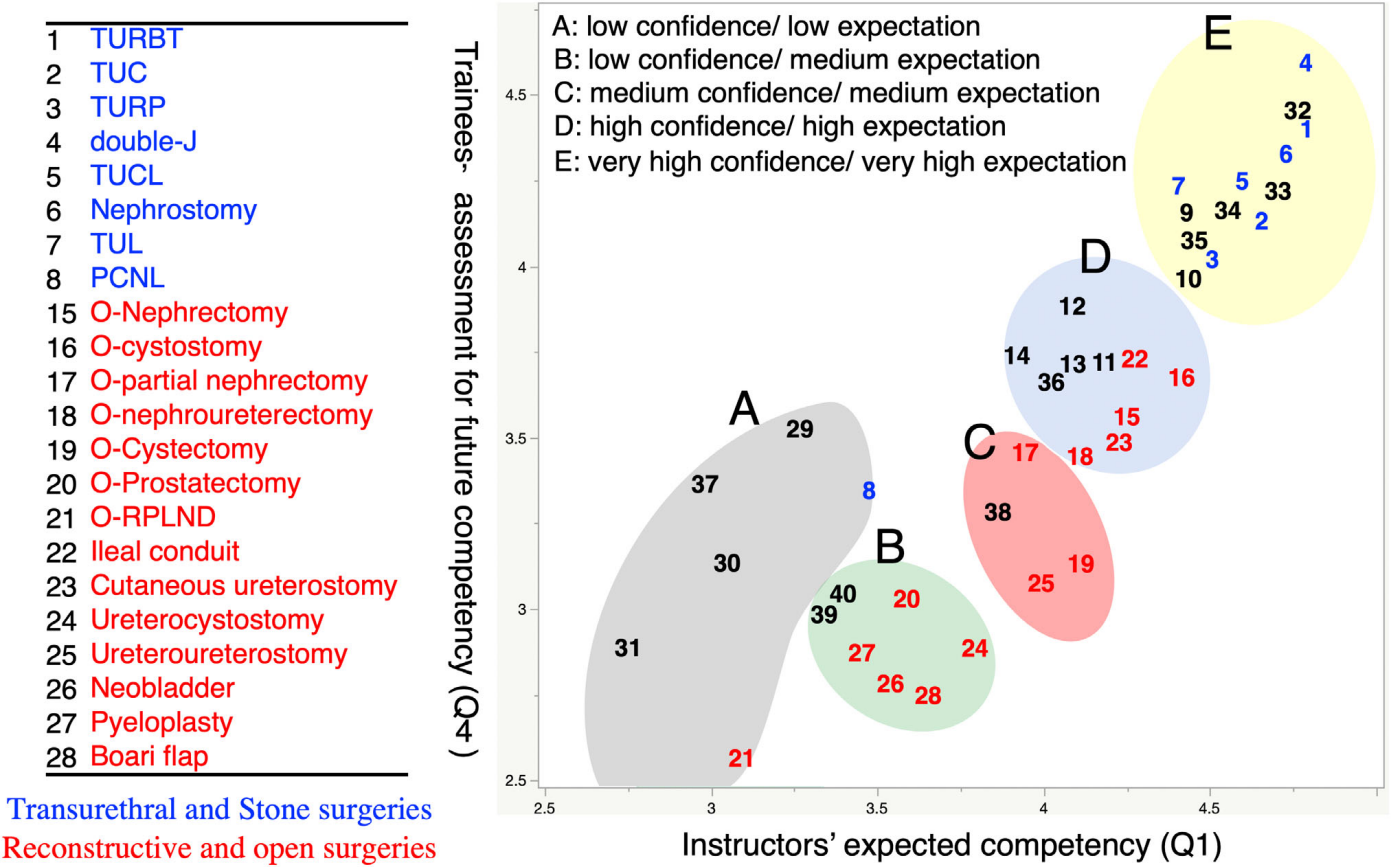
Analysis of perception gap between instructors' expectation of skill acquisition and trainees' self‐confidence. Procedures were categorized into 5 clusters [Cluster A (gray circle): low trainees' future confidence/low instructors' expectation, Cluster B (green circle): low trainees' future confidence/medium instructors' expectation, Cluster C (pink circle): medium trainees' future confidence/medium instructors' expectation, Cluster D (blue circle): high trainees' future confidence/high instructors' expectation, Cluster E (yellow circle): very high trainees' future confidence/very high instructors' expectation]. For example, ureterocystostomy (No. 24), neobladder (No. 26), pyeloplasty (No. 27), and Boari flap (No. 28) showed a relatively large recognition gap (Cluster B). PCNL, percutaneous nephrolithotripsy; RPLND, retroperitoneal lymph node dissection; TRUBT, transurethral resection of bladder tumor; TUC, transurethral electrocoagulation; TUCL, transurethral cystolithotripsy; TUL, transurethral ureterolithotripsy; TURP, transurethral resection of prostate.

**FIGURE 4:**
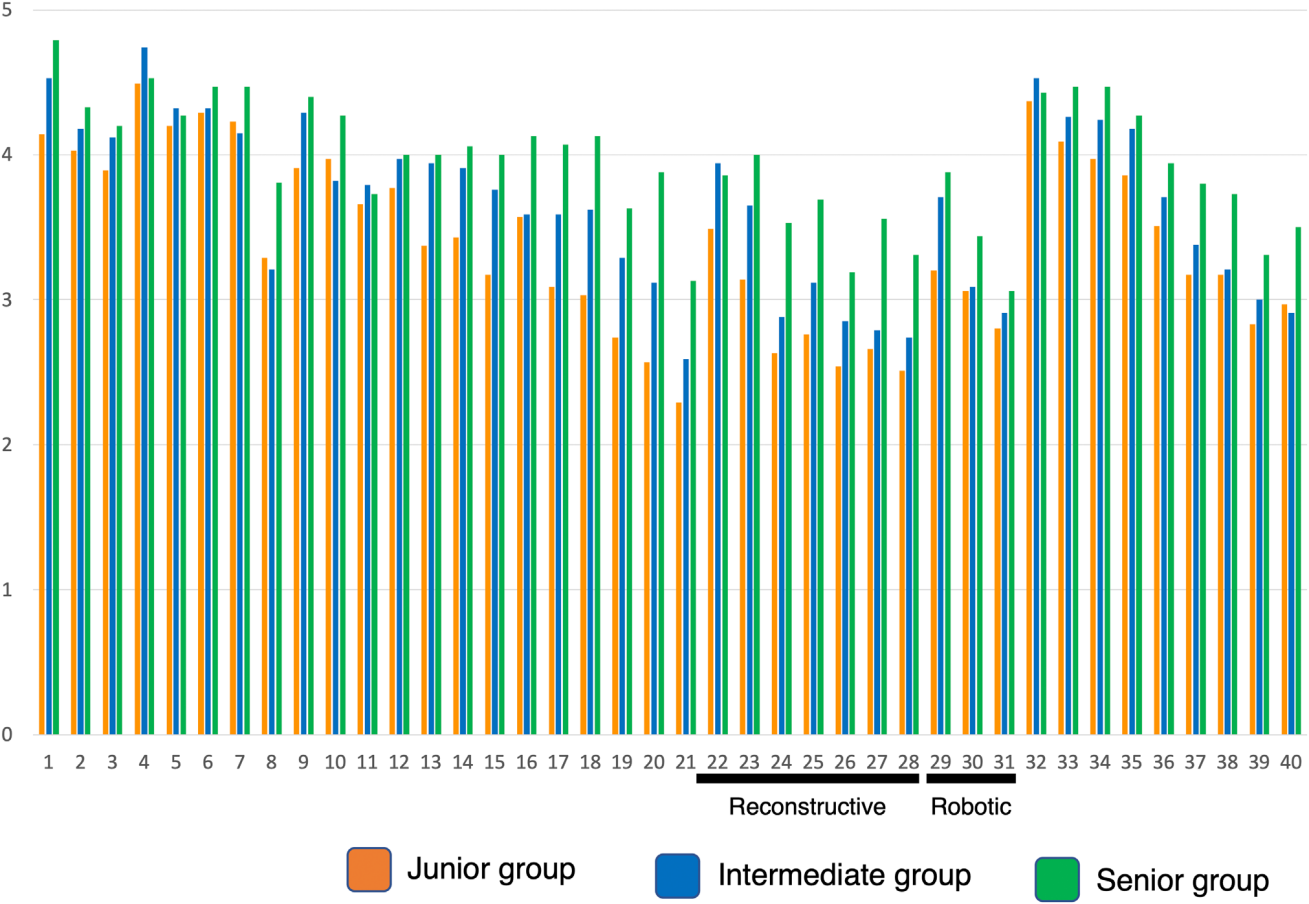
Summary of mean score of trainees' confidence for future skill acquisition divided by post‐graduate years. In ureterocystostomy (No. 24), open neobladder (No. 26), pyeloplasty (No. 27), and Boari flap (No. 28), intermediate and junior groups particularly showed low mean scores. Open retroperitoneal lymph node dissection (No. 21) and robot‐assisted radical cystectomy (No. 31) also revealed low confidence for future skill acquisition.

Figure 3 shows a summary of the recognition gap between instructors' expected competency and trainees' subjective confidence for future skill acquisition when the trainee becomes an attending urologist. Procedures were divided into 5 clusters: Cluster A (gray circle) was termed low confidence/low expectation, which means that both trainees' confidence and instructors' expectations for future skill acquisition were low. Cluster B (green circle) was termed low confidence/medium expectation, and Cluster C (pink circle) as medium confidence/medium expectation. Cluster D (blue circle) was termed high confidence/high expectation, and Cluster E (yellow circle) as very high confidence/very high expectation. Overall, there was no procedure with a very large gap between trainees' confidence and instructors' expectation for future skill acquisition. Regarding the details of each procedure, transurethral and stone (blue letters) surgeries were included in Cluster E, other than PCNL (Cluster A, No. 8). Reconstructive and open surgeries (red letters) were distributed broadly among Clusters B, C, and D. In Cluster B, ureterocystostomy (No. 24), open NB (No. 26), pyeloplasty (No. 27), and Boari flap (No. 28) revealed a relatively large recognition gap. Figure 4 shows trainees' confidence for future skill acquisition divided by PGY. In these procedures, junior and intermediate groups particularly showed low mean scores. Open RPLND and RARC also revealed low future confidence for future skill acquisition.

### Analysis 4: Instructors' versus trainees' assessment of potential risk if the procedure is performed by an inexperienced surgeon

**FIGURE 5:**
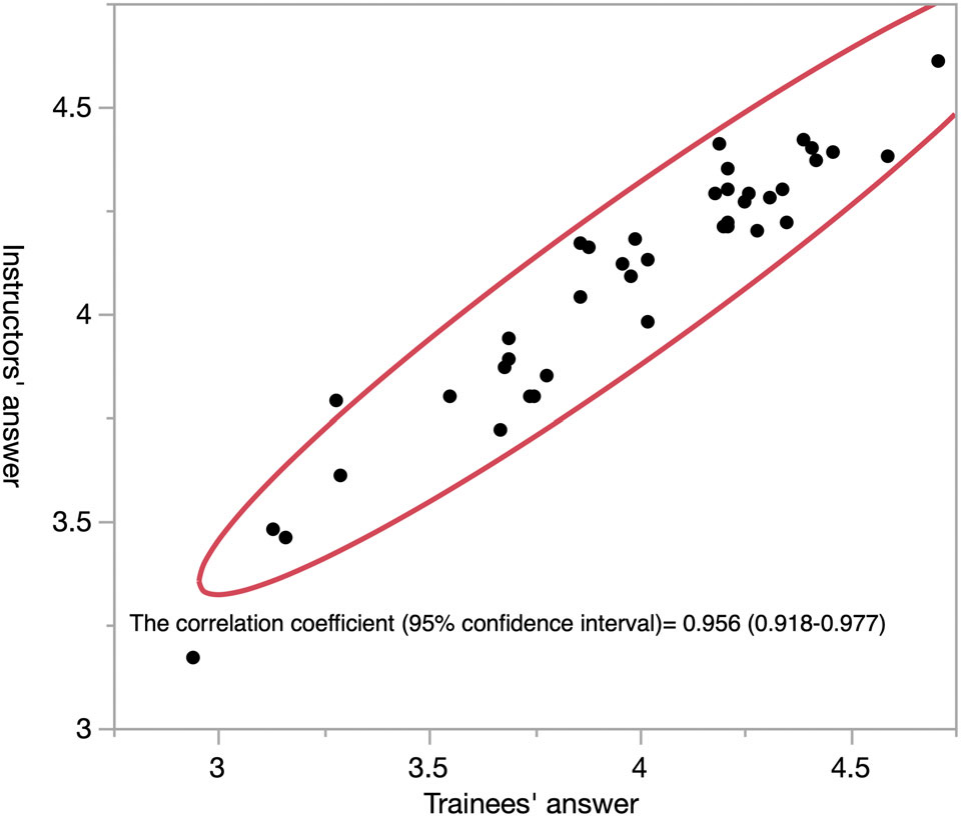
Scatter plot of potential risk assessment between trainees and instructors. The correlation coefficient was 0.956 (95% confidence interval: 0.918–0.977), showing that they generally shared similar views on the potential risk of each procedure.

Figure 5 shows a scatter plot of potential risk assessment between trainees and instructors, if each procedure is performed by an inexperienced surgeon. The correlation coefficient was 0.956 (95% confidence interval: 0.918–0.977), showing that they generally shared similar views on the potential risk of each procedure.

## DISCUSSION

Because of the growing number of surgical procedures for trainees to learn during the post‐graduate training period, such as laparoscopic and robotic surgeries, as well as a reduced surgical volume of open surgeries, we hypothesized that there may be a gap between trainees' subjective competencies and the competencies expected by instructors in several core urologic surgical procedures. In the present study, we defined doctors with less than 15 years of clinical experience as “trainees” and collected data on the current status of urological surgical education. Although some readers may argue against our definition, we considered that more than 10 years of clinical experience may be necessary to become a truly independent urologic surgeon and aimed to determine the needs for future surgical education. Thanks to the collaborative attitudes and two email reminders, we achieved a very high response rate of 96%.

Regarding the caseloads, many of the procedures were gradually experienced according to the years of clinical practice (Figure 1). As we expected, trainees started transurethral (No. 1–3) and stone (No. 4–7) surgeries in the early phase of training periods, other than PCNL (No. 8). In terms of reconstructive (No. 22–28) and robotic (No. 29–31) surgeries, the senior groups still had limited caseloads, other than ileal conduit (No. 22). In terms of open surgeries (No. 15–21), the intermediate and senior groups gradually accumulated surgical experience (No. 15–20), other than open RPLND (No. 21). Overall, less previous caseloads reflected the low subjective competency at present in the corresponding procedures (Figure 2, e.g., No. 8, 21, 26–31, 40). Several procedures still showed low‐level confidence for future skill acquisition, while instructors expected trainees to gain competency. Regarding the potential risk of each surgical procedure, trainees and instructors shared similar views on the risk.

To date, many cases have moved from an open procedure to minimum invasive approach, and, similar to Western countries, robotic surgery has now become more prevalent, such as RARP, RAPN, or RARC, in Japan. In the present study, among the 3 university and 37 teaching hospitals, 19 hospitals had Da‐Vinci surgical robots. While we observed low caseloads and a low subjective competency level in robotic surgery, a similar trend was observed after residency in Western countries.^4^, ^5^ In our experience, because young trainees have a strong interest in robotic surgery, we need to develop a standardized training curriculum for robotic surgery, which may be imperative for skill education, patient safety, and recruitment of future urologic surgeons. Thanks to the dual console if available, numerous experts' surgical movies available via websites, live surgery by streaming robotic surgeries from leading robotic centers,^7^ or simulation training such as virtual reality simulators and cadaveric training,^8^, ^9^ we consider that robot‐assisted surgery has inherent advantages for surgical training.

At present, the decline of open surgical experience has become a common issue in surgical specialties.^10^, ^11^, ^12^ As we expected, trainees showed low subjective competencies and, furthermore, low confidence for future skill acquisition in several open and reconstructive procedures, such as open cystectomy, ureteroureterostomy, and ureterocystostomy, while instructors expected trainees to be able to perform these procedures independently when they became an attending doctor. Regarding open procedures, we also think that skills, experience, and competency to complete open surgery are necessary, because open conversion is required when a minimum invasive approach cannot be safely completed due to severe adhesion or massive bleeding. In terms of reconstructive techniques, iatrogenic ureteric injuries can occur during both open and minimum invasive surgery including urologic and gynecologic procedures,^13^, ^14^ and urologists have to manage this complication immediately during the surgery, or later if ureteric injury has gone unnoticed during the surgery. Although the surgical volume of open surgery is gradually decreasing, we should expose trainees to a broad range of open and reconstructive procedures, as many as possible, utilizing real experiences of clinical cases or simulation‐based education including cadaveric training,^15^ or developing video libraries. Ideally, trainees should learn the technical basics of these reconstructive procedures, both with open and minimum invasive approaches.

Recently, perioperative teaching and feedback are considered to be imperative for surgical education.^16^, ^17^ For example, Roberts et al. developed the BID model (briefing, intraoperative teaching, and debriefing).^18^ Popat et al. also reported a similar model of education time out (before surgery) and debrief between faculty members and trainees.^19^ Without a doubt, fostering a good culture and giving sufficient feedback to the next generation of surgeons should be pivotal parts of surgical education.

This study had several limitations. First, competency was based on subjective assessments by the trainees and not an objective assessment of their surgical skills. Thus, it may not reflect the true surgical skills of the trainees. Video review by multiple instructors or simulation trainings including cadaveric surgical training may be another way to perceive trainees' skill competency. Second, the present study was a regional survey among Japanese urologists in Hokkaido Prefecture, and so our observation may be biased from this area. The number of urologic surgeons and surgical volume varies depending on the region, such as between metropolitan and rural areas. The community size should influence clinical practice and the current training situation. The surgical volume, variation of procedures, and policy of each training hospital should also influence the training during the rotation periods. However, we consider that the decreasing number of open surgery caseloads may be a common issue in surgical education. Third, instructors' expected competency could be affected by each instructor's own skill acquisition level. Fourth, during the authors' discussion, we considered that more than 10 years of clinical experience may be necessary to become an independent urologic surgeon, and we decided to use our cutoff of 15 years of clinical experience for data collection. Since the JUA has the board system of certified doctors, this definition could have been another option for categorization of trainees and instructors. Nevertheless, we believe that our observation of the perception gap between trainees and instructors could give several clues for future improvements of surgical training in urology, not only in Japan but also in other countries.

## AUTHOR CONTRIBUTIONS

**Kanta Hori:** data curation; format analysis; visualization; writing original draft. **Takashige Abe:** conceptualization; project administration; writing review & editing. **Noriyuki Abe:** data curation. **Junya Abe:** data curation. **Kazufumi Okada:** format analysis. **Keita Takahashi:** format analysis. **Shigeru Harada:** data curation. **Jun Furumido:** data curation. **Sachiyo Murai:** data curation. **Masafumi Kon:** conceptualization; data curation. **Kohei Hashimoto:** data curation. **Naoya Masumori:** supervision. **Hidehiro Kakizaki:** supervision. **Nobuo Shinohara:** supervision.

## CONFLICT OF INTEREST STATEMENT

The authors declare no conflict of interest.

## APPROVAL OF THE RESEARCH PROTOCOL BY AN INSTITUTIONAL REVIEWER BOARD

This study was reviewed and approved by the institutional review board of the Hokkaido University Graduate School of Medicine (Approval ID: I 20‐035).

## INFORMED CONSENT

All participants provided written informed consent with guarantees of confidentiality.

## REGISTRY AND THE REGISTRATION NO. OF THE STUDY/TRIAL

Not applicable.

## ANIMAL STUDIES

Not applicable.

## Supporting information


Table S1.

Table S2.

Table S3.

Table S4.

Table S5.
