# Supplementary material for: Gap analysis between trainees' subjective competencies and the competencies expected by instructors in urology: A need assessment survey in Japan
Source: Int J Urol. 2024 Feb 17;31(6):653–61. doi: 10.1111/iju.15430 (PMC11524097; doi:10.1111/iju.15430)
Supplement: Supplementary file 1 — Table S1. [file IJU-31-653-s004.docx]

# **Supplementary Table 1.** details of the Trainees’ specific questions (English translation version)

Questions about yourself

1. Sex

○ Male

○ Female

○ Other/Prefer not to answer

1. Age


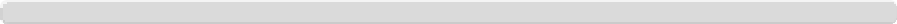

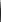

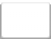

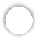

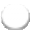


0 years old

100 years old

1. E-mail
2. Current affiliation
3. Year of MD completion
4. Years of experience in urology


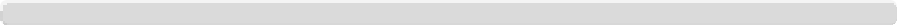

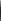

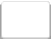

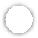

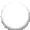


0 years

50 years

1. Are you board-certified as a urology specialist by the Japanese Urological Association?

○ Yes

○ No

1. Are you board-certified as a urology instructor by the Japanese Urological Association?

○ Yes

○ No

1. Areas of specialization (check all that apply)

🞎 Pediatric urology

🞎 Female urology

🞎 Kidney transplant

🞎 Neurourology

🞎 Urologic oncology

🞎 Urinary tract stones

🞎 Andrology

🞎 Infertility

🞎 Have yet to decide

🞎 None

🞎 Others (please specify)

1. Have you completed certification by the Japanese Society of Endourology and Robotics for laparoscopic surgery technique?

○ Yes

○ No

1. Are you certified for robot-assisted surgery (e.g., as a ‘console surgeon’)?

○ Yes, I am a certified proctor.

○ Yes, I am a certified console surgeon.

○ No

1. In urology, I am board-certified as (check all that apply):

🞎 Oncologist

🞎 Pediatric urologist

🞎 Kidney transplant specialist

🞎 Dialysis specialist (certified by the Japanese Society for Dialysis Therapy)

🞎 Continence specialist (certified by the Japanese Continence Society)

🞎 None

🞎 Others (please specify)

1. What is the total number of open cases in which you were involved as the primary surgeon?

○ 0

○ 1-10

○ 11-50

○ 51-100

○ 101-500

○ 501 and above

1. What is the total number of laparoscopic cases in which you were involved as the primary surgeon?

○ 0

○ 1-10

○ 11-50

○ 51-100

○ 101-500

○ 501 and above

1. What is the total number of robotic cases in which you were involved as the primary surgeon?

○ 0

○ 1-10

○ 11-50

○ 51-100

○ 101-500

○ 501 and above

1. What is the total number of transurethral cases in which you were involved as the primary surgeon (regardless of the surgical technique, e.g., TUR-BT, TUR-P, urethroscopy, TUL)?

○ 0

○ 1-10

○ 11-50

○ 51-100

○ 101-500

○ 501 and above

# 1. Transurethral removal of bladder tumor (TURBT)

*What is the total number of TURBT cases in which you were involved as a surgeon?

○ 0

○ 1-10

○ 11-50

○ 51-100

○ 101-500

○ 501 and above

*Subjective assessment of your autonomy (Dreyfus scale)

| ○ 1. I am comfortable as an observer and/or an assistant  (Primary instructors perform most of the procedure and related techniques with explanations while I observe and assist as needed) | ○ 4. I can perform procedures independently  (I can perform the procedure independently and safely under the guidance of my instructors) |
| --- | --- |
| ○ 2. I need active assistance from my instructors  (I can perform the procedure under my instructors’ guidance and active assistance) | ○ 5. I can perform procedures independently  (I can perform the procedure safely without the guidance of my instructors or supervisors) |
| ○ 3. I sometimes need advice and assistance from my instructors  (I can perform most of the procedure myself but sometimes need advice and assistance from my instructors) |  |

*This procedure may cause significant complications and risks to patients if performed by an inexperienced surgeon

| ○ 1. Strongly disagree | ○ 4. Agree |
| --- | --- |
| ○ 2. Disagree | ○ 5. Strongly agree |
| ○ 3. Neither agree nor disagree |  |

Based on your experience as a trainee, do you think you will be able to acquire the techniques required for the procedure?

• If you have not yet obtained a certification as an instructor, please think of when you successfully obtain your certification as your endpoint.

• If you have already obtained a certification as an instructor but believe that you have not reached the level of competency, please think of how you might improve with additional training.

| ○ 1. Strongly disagree | ○ 4. Agree |
| --- | --- |
| ○ 2. Disagree | ○ 5. Strongly agree |
| ○ 3. Neither agree nor disagree |  |

*The same questions were asked for the remaining 39 procedures listed in Table 1.
