# Supplementary material for: Gap analysis between trainees' subjective competencies and the competencies expected by instructors in urology: A need assessment survey in Japan
Source: Int J Urol. 2024 Feb 17;31(6):653–61. doi: 10.1111/iju.15430 (PMC11524097; doi:10.1111/iju.15430)
Supplement: Supplementary file 3 — Table S3. [file IJU-31-653-s001.pdf]

修練医各論

Supplementary Table 3. details of the Trainees' specific questions (original Japanese version)

あなた自身についての質問

1. 性別

- ○ 男
- ○ 女
- ○ その他または回答しない

2. あなたの年齢をお教えください

0歳 100歳

3. メールアドレス

4. 現在所属されている御施設名

5. 医学部卒業年（西暦）

6. 泌尿器科経験年数

0年 50年

7. 日本泌尿器科学会・泌尿器科専門医資格取得の有無

- ○ 取得あり
- ○ 取得なし

8. 日本泌尿器科学会・泌尿器科指導医資格取得の有無

- ○ 取得あり
- ○ 取得なし

9. ご専門の泌尿器科の領域（複数回答可）

- ☐ 小児泌尿器科
- ☐ 女性泌尿器科
- ☐ 腎移植
- ☐ 神経泌尿器科
- ☐ 泌尿器科腫瘍
- ☐ 尿路結石
- ☐ アンドロロジー
- ☐ 不妊治療
- ☐ 検討中
- ☐ なし
- ☐ その他 (具体的に)

10. 日本泌尿器内視鏡学会腹腔鏡手術技術認定取得の有無

- ◯ 取得あり
- ◯ 取得なし

11. ロボット支援手術認定資格(コンソールサージョン資格)の有無

- ◯ プロクターの資格を有する
- ◯ コンソールサージョンの資格を有する
- ◯ いずれの資格も持たない

12. 取得されている泌尿器科領域の認定資格 (複数回答可)

- ☐ がん治療認定医
- ☐ 小児泌尿器科学会認定医
- ☐ 腎移植認定医
- ☐ 透析医学会専門医
- ☐ 排尿機能学会認定医
- ☐ なし
- ☐ その他 (具体的に)

13. これまでに<u>主たる術者で参加した</u>開腹手術件数の概数

- ◯ 0件
- ◯ 1-10件
- ◯ 11-50件
- ◯ 51件−100件
- ◯ 101−500件
- ◯ 501件以上

14. これまでに<u>主たる術者で参加した</u>腹腔鏡手術件数の概数

- ◯ 0件
- ◯ 1-10件
- ◯ 11-50件
- ◯ 51件−100件
- ◯ 101−500件
- ◯ 501件以上

15. これまでに<u>主たる術者で参加した</u>ロボット手術件数の概数

- ◯ 0件
- ◯ 1-10件
- ◯ 11-50件
- ◯ 51件−100件
- ◯ 101−500件
- ◯ 501件以上

16. これまでに<u>主たる術者で参加した</u>経尿道的手術の総数、TUR-Bt、TUR-P、尿管鏡、TUL等術式を問わず

- ◯ 0件
- ◯ 1-10件
- ◯ 11-50件
- ◯ 51件−100件
- ◯ 101−500件
- ◯ 501件以上

1. 経尿道的膀胱腫瘍切除術

＊これまでに術者として執刀された件数

- ◯ 0件
- ◯ 1-10件
- ◯ 11-50件
- ◯ 51-100件
- ◯ 101-500件
- ◯ 501件以上

＊現在の自立度の自己評価(Zwisch model)

- ◯ 1. 観察・助手のみ
  指導医が大半の手術手順および手技を解説しながら行い、修練医は観察および助手を務めるレベル
- ◯ 2. 指導医の積極的な介助が必要
  修練医は指導医の積極的な介助ならびに指導の下で手術を行えるレベル
- ◯ 3. ときに指導医の助言や介助が必要
  修練医が手術の大半を自ら行えるが、時に指導医の助言や介助を必要とするレベル
- ◯ 4. 自立して手術を行える
  指導医の監視下ではあるが、修練医が手術を自立して安全に行えるレベル
- ◯ 5. 自立して手術を行える
  指導医・上級医の監視なしで安全に行えるレベル

＊本術式は、未熟な医師が執刀した場合、患者に大きな不利益・危険を生じる可能性がある。

- ◯ 1. まったくそう思わない
- ◯ 2. そう思わない
- ◯ 3. 平均
- ◯ 4. そう思う
- ◯ 5. 強くそう思う

これまでの研修状況から予想すると、本術式の習得は可能でしょうか？
・指導医取得前の先生は、指導医取得時点を目安に予想してください。
・指導医取得後の先生では、もし本術式を自立して行うレベルに到達していないと思われるなら、今後の研修による上積みを予想してお答えください。

- ◯ 1. まったくそう思わない
- ◯ 2. そう思わない
- ◯ 3. どちらとも言えない
- ◯ 4. そう思う
- ◯ 5. 強くそう思う

※以降、Table1に記載した残りの39術式に関して同様の形式で回答を回収した。
