# Supplementary material for: Gap analysis between trainees' subjective competencies and the competencies expected by instructors in urology: A need assessment survey in Japan
Source: Int J Urol. 2024 Feb 17;31(6):653–61. doi: 10.1111/iju.15430 (PMC11524097; doi:10.1111/iju.15430)
Supplement: Supplementary file 4 — Table S4. [file IJU-31-653-s002.pdf]

指導医各論

Supplementary Table 4. details of the Instructors' specific questions (original Japanese version)

あなた自身についての質問

1. 性別
   - 男
   - 女
   - 回答しない

2. あなたの年齢をお教えください

   0歳　　　100歳

3. メールアドレス

4. 現在所属されている御施設名

5. 医学部卒業年（西暦）

6. 泌尿器科経験年数

   0年　　　50年

7. 日本泌尿器科学会・泌尿器科専門医資格取得の有無
   - 取得あり
   - 取得なし

8. 日本泌尿器科学会・泌尿器科指導医資格取得の有無
   - 取得あり
   - 取得なし

9. ご専門の泌尿器科の領域（複数回答可）

- [ ] 小児泌尿器科
- [ ] 女性泌尿器科
- [ ] 腎移植
- [ ] 神経泌尿器科
- [ ] 泌尿器科腫瘍
- [ ] 尿路結石
- [ ] アンドロロジー
- [ ] 不妊治療
- [ ] なし
- [ ] その他 (具体的に)

10. 日本泌尿器内視鏡学会腹腔鏡手術技術認定取得の有無

- ○ 取得あり
- ○ 取得なし

11. 10で無の場合、御施設での日本泌尿器内視鏡学会腹腔鏡手術技術認定取得者の有無

- ○ あり
- ○ なし

12. ロボット支援手術認定資格(コンソールサージョン資格)の有無

- ○ プロクターの資格を有する
- ○ コンソールサージョンの資格を有する
- ○ いずれの資格も持たない

13. 取得されている泌尿器科領域の認定資格 (複数回答可)

- [ ] がん治療認定医
- [ ] 小児泌尿器科学会認定医
- [ ] 腎移植認定医
- [ ] 透析医学会専門医
- [ ] 排尿機能学会認定医
- [ ] なし
- [ ] その他 (具体的に)

14. 御施設におけるあなたの主な役割 (複数回答可)

- [ ] 教授、部長など泌尿器科の責任者
- [ ] 院内研修医教育の責任者
- [ ] 泌尿器科修練医の指導に携わっている泌尿器科スタッフの一員
- [ ] シュミレーションセンター・スキルスラボの責任者
- [ ] その他 (具体的に)

15. これまでに主たる術者・指導的立場で参加した開腹手術の総数(術式を問わず)

- 0件
- 1-10件
- 11-50件
- 51件−100件
- 101−500件
- 501件以上

16. これまでに主たる術者・指導的立場で参加した腹腔鏡手術の総数(術式を問わず)

- 0件
- 1-10件
- 11-50件
- 51件−100件
- 101−500件
- 501件以上

17. これまでに主たる術者・指導的立場で参加したロボット手術の総数(術式を問わず)

- 0件
- 1-10件
- 11-50件
- 51件−100件
- 101−500件
- 501件以上

18. これまでに主たる術者・指導的立場で参加した経尿道的手術の総数、TUR-Bt、TUR-P、尿管鏡、TUL等術式を問わず

- 0件
- 1-10件
- 11-50件
- 51件−100件
- 101−500件
- 501件以上

1. 経尿道的膀胱腫瘍切除術

* 本術式は、日本泌尿器科学会指導医資格取得の時点で、独立した術者として執刀できる必要がある

- 1.全くそう思わない
- 2.そう思わない
- 3.どちらとも言えない
- 4.そう思う
- 5.強くそう思う

* 本術式は、専門分野の泌尿器科医師が執刀できる必要がある

- 1.全くそう思わない
- 2.そう思わない
- 3.どちらとも言えない
- 4.そう思う
- 5.強くそう思う

* 本術式は、未熟な医師が執刀した場合、患者に大きな不利益・危険を生じる可能性がある

- 1.全くそう思わない
- 2.そう思わない
- 3.平均
- 4.そう思う
- 5.強くそう思う

* 現在の施設の規模/指導医の専門の点から、本術式を教育できる

- 1.全くそう思わない
- 2.そう思わない
- 3.どちらとも言えない
- 4.そう思う
- 5.強くそう思う

※以降、Table1に記載した残りの39術式に関して同様の形式で回答を回収した。
